# Visualizing DNA single- and double-strand breaks in the Flash comet assay by DNA polymerase-assisted end-labelling

**DOI:** 10.1093/nar/gkae009

**Published:** 2024-01-23

**Authors:** Erik Bivehed, Björn Hellman, Leonie Wenson, Bo Stenerlöw, Ola Söderberg, Johan Heldin

**Affiliations:** Department of Pharmaceutical Biosciences, Uppsala University, Biomedical Centre, Uppsala SE-751 24, Sweden; Department of Pharmaceutical Biosciences, Uppsala University, Biomedical Centre, Uppsala SE-751 24, Sweden; Department of Pharmaceutical Biosciences, Uppsala University, Biomedical Centre, Uppsala SE-751 24, Sweden; Department of Immunology, Genetics and Pathology, Uppsala University, Rudbeck Laboratory, Uppsala SE-751 85, Sweden; Department of Pharmaceutical Biosciences, Uppsala University, Biomedical Centre, Uppsala SE-751 24, Sweden; Department of Pharmaceutical Biosciences, Uppsala University, Biomedical Centre, Uppsala SE-751 24, Sweden

## Abstract

In the comet assay, tails are formed after single-cell gel electrophoresis if the cells have been exposed to genotoxic agents. These tails include a mixture of both DNA single-strand breaks (SSBs) and double-strand breaks (DSBs). However, these two types of strand breaks cannot be distinguished using comet assay protocols with conventional DNA stains. Since DSBs are more problematic for the cells, it would be useful if the SSBs and DSBs could be differentially identified in the same comet. In order to be able to distinguish between SSBs and DSBs, we designed a protocol for polymerase-assisted DNA damage analysis (PADDA) to be used in combination with the Flash comet protocol, or on fixed cells. By using DNA polymerase I to label SSBs and terminal deoxynucleotidyl transferase to label DSBs with fluorophore-labelled nucleotides. Herein, TK6-cells or HaCat cells were exposed to either hydrogen peroxide (H_2_O_2_), ionising radiation (X-rays) or DNA cutting enzymes, and then subjected to a comet protocol followed by PADDA. PADDA offers a wider detection range, unveiling previously undetected DNA strand breaks.

## Graphical Abstract

**Graphical Abstract:**
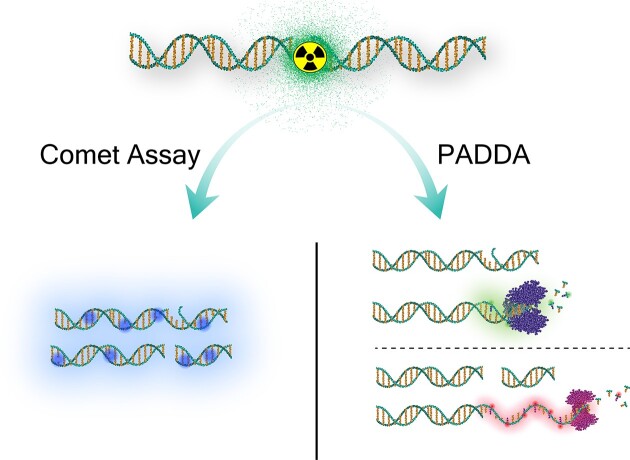

## Introduction

During the last three decades, the level of DNA damage in individual cells has been evaluated using single-cell gel electrophoresis under neutral (1,2) or alkaline conditions (3,4). The latter version, also known as the comet assay, has been used when evaluating the potential genotoxicity of chemicals, to study DNA-repair, and for biomonitoring purposes (5–9). Most protocols for single-cell gel electrophoresis both under neutral (1) and alkaline conditions (3), typically result in comets with or without tails. Whereas the comet head represents the nucleoid, the tail represents DNA loops and/or fragments extended from the head during the electrophoresis. High levels of DNA strand breaks usually result in prominent tails.

During the course of single-cell gel electrophoresis under alkaline conditions, tails are formed, which can include both SSBs and DSBs. The assay can also be performed with the addition of pyrimidine glycosylase or endonuclease III before the electrophoresis, to capture oxidative DNA damage (10,11). It is well known that many different types of genotoxic agents increase the level of DNA strand breaks, and it is equally well known that SSBs are constantly formed in our cells during natural processes. For example, during the replication and repair of DNA, and as a result of various metabolic processes (12,13). Therefore, it can be assumed that double-strand breaks (DSBs) are much less abundant than single-strand breaks (SSBs) in a comet tail. A drastic increase in SSBs without concomitant cytotoxicity is indeed a warning signal if not repaired correctly (which they normally are), but a corresponding increase in DSBs is more problematic, at least when it comes to the evaluation of an increase in DNA strand breaks following from an exposure to a genotoxic agent. It would therefore be beneficial if it was possible to distinguish between SSBs and DSBs in comet tails when evaluating an induced increase in the level of DNA damage. It is often assumed that a comet assay performed under more neutral conditions (pH 10) will capture DSBs selectively, but as we recently have shown when investigating the DNA integrity at different pH (10, 12.5 or >13), this is not the case (14). Also, the so-called neutral version of the comet assay will show a mixture of SSBs and DSBs, even if the DSBs will dominate.

Recently we also described a new protocol for the comet assay (the Flash comet), enabling the analysis and quantification of DNA strand breaks using a low conductivity electrophoresis solution based on LiOH at pH 12.8, instead of NaOH at pH >13 (15,16). The objective of the present paper was to establish a protocol to distinguish between SSBs and DSBs in the Flash comet heads and tails. In order to do so, we designed a protocol for polymerase-assisted DNA damage analysis (PADDA) to visualize SSBs and DSBs (17) in the comet assay. In order to label SSBs we utilized *E. Coli* DNA polymerase I (Pol I), which will extend free 3′-OH groups in a template-dependent manner, and to label DSBs we used terminal deoxynucleotidyl transferase (TdT), which will extend free 3′-OH groups in a template-independent manner. In the Comet-PADDA, these two enzymes were used sequentially after the cells had been lysed and subjected to electrophoresis using the Flash comet protocol, or fixed using ethanol fixation. The two different polymerases incorporate nucleotides labelled with different fluorophores, in order to generate different fluorescent extension products downstream of SSBs and DSBs.

Using this strategy allowed us to selectively visualize both SSBs and DSBs in the comet heads and tails. Even if other strategies have been described to monitor both SSBs and DSBs in modified protocols for the comet assay, including the so called two-tailed comet assay (18–21). However, not only our own work (14), but also findings reported by other research groups (22–24) have shown that SSBs and DSBs cannot be clearly distinguished by using the comet assay, neither under neutral, nor under alkaline conditions, when used in combination with conventional staining techniques. Therefore, the herein described approach has, to the best of our knowledge, never been used before to distinguish between SSBs and DSBs in comets after alkaline single-cell gel electrophoresis.

The chemical (H_2_O_2_), physical (ionizing radiation from X-rays) and biological (a nickase, Nt.BsmAI and the restriction enzyme RsaI) exposures included in the present study were chosen because they are all known to induce DNA strand breaks. H_2_O_2_ is mainly known to induce SSBs, but has also been shown to induce DSBs directly or indirectly, e.g. at high concentrations, in cells during the S-phase and in the presence of histidine (15,25–28). Whereas ionizing radiation is known to induce both SSBs and DSBs (27,29–31), nickases such as Nt.BsmAI are utilized to specifically induce SSBs (32,33), and a restriction enzyme such as RsaI, to induce DSBs (34). By using the Flash comet protocol in combination with the PADDA, we found that all four exposures induced DNA strand breaks in the TK6-cells, and that it was possible to discriminate between SSBs and DSBs in the comets. Interestingly, we also observed that H_2_O_2_, and ionizing radiation induced different patterns of DNA strand breaks in the comet tails.

## Materials and methods

### Reagents

Enzymes: In the present study *E. coli* DNA polymerase I (Pol I, M0209) and Terminal deoxynucleotidyl transferase (TdT, M0315) were used to detect SSBs and DSBs, respectively. For single-stranded nicking of the genomic DNA, the nickase Nt.BsmAI (R012) was used and for double-strand breaks of the genomic DNA the restriction enzyme RsaI (R0167) was used. All enzymes were acquired from New England Biolabs (NEB, USA).

Fluorescent stains and fluorescently labelled nucleotides: SYBR Gold (Thermofischer Scientific, UK), Hoechst 33342 (Thermofischer Scientific, UK), propidium iodide (Merck, Germany), acridine orange (Merck, Germany), and dUTP labelled with Alexa 488, Alexa 555 or Alexa 647 (NU-803-XX-AF488/AF555/AF647, Jena bioscience, Germany).

Other chemicals and reagents: Unlabelled nucleotides (R0182, Thermofischer Scientific), BSA (NEB) and hydrogen peroxide (H_2_O_2_; Merck, Germany). Unless specifically indicated, all other chemicals and reagents used in the present study were of analytical grade and purchased from Merck (Germany).

### Biological resources

Cell line and culture medium: Human lymphoblastoid TK6-cells was purchased from ATCC (catalogue number CRL-8015, USA) and kept frozen at –150°C until use. The cells were cultivated in Gibco^TM^ RPMI 1640 medium (Thermofischer Scientific, UK) supplemented with 10% fetal bovine serum (FBS, Biological Industries, Israel), 1% Gibco™ PenStrep (10 000 U penicillin/ml and 10 000 μg/ml streptomycin; Thermofischer Scientific, UK), hereafter referred to as the growth medium. After an ampoule of frozen cells was thawed, the cells were maintained in suspension in a temperature-controlled atmosphere at 37°C with 5% CO_2_ until the experiments were performed on cultivation day seven. HaCat cells (kind gift from professor Aristidis Moustakas, Uppsala University) were cultured in DMEM supplemented with 10% fetal bovine serum at temperature-controlled atmosphere at 37°C with 5% CO_2_ until the experiments were performed.

### Flash comet

As previously described in detail (35), with some minor modifications, the cells were washed with PBS (pH 7.4) at 4°C after the exposure. The cells were then embedded in 0.6% low-melting point agarose (Thermofischer Scientific, UK) and casted on microscope slides before subjected to lysis for 1 h on ice, using a solution of 2.5 M NaCl, 100 mM Na_2_-EDTA, 10 mM Tris, 1% Triton X-100 and 5% DMSO (pH 8.5 adjusted with NaOH). The slides were then transferred to an electrophoresis tank (Merck, Germany) and immersed in 1700 ml of a low conductivity electrophoresis solution (30 mM LiOH, pH 12.5). The DNA was unwound at 4°C during 2.5 min, before they were subjected to electrophoresis for 1 minute at 150 V (5 V/cm) in the same tank. The slides were then neutralized with 0.4 M Tris–HCl (pH 7.5), and fixated by ethanol (70:95:100%; using 2 min per step). After that, the slides were dried at room temperature in a hood, and stored in a sealed box until they were stained with conventional fluorescent dyes (in the staining experiment and the conventional Flash comet), or subjected to SSB and DSB labelling using the PADDA protocol (described below).

### Polymerase-assisted DNA damage analysis (PADDA)

For SSB detection, the dehydrated slides were rehydrated and incubated with 0.25 U/μl DNA polymerase I (Pol I) diluted in 2x NEBbuffer 2 (NEB), 0.5 mg/ml BSA, 0.2 mM dATP/dGTP/dCTP, 0.16 mM dTTP, 0.04 mM Aminoallyl-dUTP-XX-AF488 (or AF555) for 90 min at 37°C in a moisture chamber. Thereafter, the slides were subjected to a series of washes: 3 times quickly rinsed with H_2_O, 2 × 5 min with TBS–tween-20 (150 mM NaCl, 50 mM Tris–HCl, pH 7.6 and 0.05% tween-20) and finally 2 × 5 min with TBS. To detect DSBs the slides were thereafter incubated with 1.6 U/μl Terminal deoxynucleotidyl transferase (TdT) diluted in 2× TdT buffer (NEB), 0.5 mg/ml BSA, 0.18 mM dTTP, 0.02 mM aminoallyl-dUTP-XX-AF555 (or AF647) and 1 mM CoCl_2_ (NEB) for 90 min at 37°C in a moisture chamber. Afterwards, the slides were again subjected to a series of washes: 3 times quickly rinsed with H_2_O, 5 min with 10 μg/ml Hoechst 33342 diluted in TBS-tween, 5 min with TBS–tween and finally 2 × 5 min with TBS. The slides were mounted with ProLong Glass antifade (Thermofisher scientific) and let to cure overnight before being imaged. All experiments were conducted three independent times and at least five images were acquired per replicate. For experiments where quantification was performed, enough images were captured to ensure that ≥100 number of nucleoids could be analysed per replicate.

### DNA staining experiment

Four different conventionally used fluorescent dyes in the comet assay were tested: SYBR Gold (1:10000), Hoechst 33342 (10 μg/ml), propidium iodide (0.1 mg/ml), and acridine orange (1 mg/ml). The PADDA was performed as described above, however, the fluorescent nucleotide was omitted for SSB detection, and in order not to interfere with the fluorescence emitted from the DNA stains, aminoallyl-dUTP-XX-AF647 was used in combination with TdT for the DSB detection. The staining experiment was performed on TK-6 cells that had been exposed to 50 μM H_2_O_2_ for 15 min.

### Hydrogen peroxide exposure

Aliquots of cell suspension corresponding to 1 × 10^6^ cells were transferred to test tubes containing growth medium. Exposure solutions of either vehicle (H_2_O) or H_2_O_2_ (50 μM) were added to the cells for 15 min at 37°C. After exposure, the cell suspensions were subjected to the Flash comet in order to get comets for the PADDA.

### Ionizing radiation exposure

TK6-cells were embedded in 0.6% low-melting point agarose and casted on microscope slides and kept in ice-cold PBS until the irradiation. The irradiation with X-rays was performed with an Elekta Versa HD linear accelerator at Uppsala University Hospital. The beam quality was 6 MV X-rays and the slides were placed at a water-equivalent depth of 10 cm by the use of water-equivalent plastic attenuators. Gel embedded cells on slides were irradiated with 0, 10 or 100 Gy on ice using a vertical beam. To obtain the accumulated radiation dose of 100 Gy, the cells were exposed to 10 Gy, ten times. The dose rate was approximately 5 Gy per minute. Directly after the irradiation, the cells were put in ice cold lysis buffer for 1 h and then subjected to the Flash comet in order to get comets for the PADDA, or for a conventional image analysis of comets.

### Nickase (Nt.BsmAI) and restriction enzyme (RsaI) exposure

The cells were embedded in 0.6% low-melting point agarose and casted on microscope slides and lysed for 1 h. The gel embedded nucleoids were treated with six concentrations of Nt.BsmAI (0, 0.0125, 0.125, 1.25, 12.5 and 125 mU/μl) or six concentrations of RsaI (0, 2.5, 25, 250, 2500 and 25000 μU/μl) for 1 h at 37°C. Nt.BsmAI is a nicking endonuclease that recognizes the sequence GTCTCN*N in double stranded DNA and cuts one of the strands, thus creating SSBs in the double-stranded DNA. RsaI is a restriction enzyme that recognizes the sequence GT*AC in double stranded DNA and cuts both strands, thus creating blunt DSBs in the double-stranded DNA. After the treatment, the embedded cells were subjected to electrophoresis following the Flash protocol in order to get comets for the PADDA.

### PADDA on ethanol fixed cells (on-slide)

HaCat cells were seeded in chamber slides and cultivated for 24 h. Thereafter washed once with ice-cold PBS and fixed with ice-cold ethanol (70%) for 30 min, followed by 5 min wash with 96% ethanol and thereafter the slides were stored in 99.5% ethanol at 4°C until use. To avoid inducing additional DNA damage, it is important that the cells are not allowed to dry, therefore the slides were washed with 1 × 5 min with TBS-tween (150 mM NaCl, 50 mM Tris–HCl, pH 7.6 and 0.05% tween-20) and 1 × 5 min with TBS to remove residual ethanol before starting a modified PADDA protocol. To induce SSBs and DSBs enzymatically, the slides were either subjected to no restriction enzyme, 125 mU/μl Nt.BsmAI or 25 mU/μl RsaI in 1x CutSmart buffer (NEB) and incubated for 1 h at 37°C followed by 2 × 5 min washes with TBS. For SSB detection, the slides were incubated with 0.25 U/μl Pol I diluted in 1× NEBbuffer 2 (NEB), 0.25 mg/ml BSA, 0.1 mM dATP/dGTP/dCTP, 0.08 mM dTTP, 0.02 mM aminoallyl-dUTP-XX-AF488 for 60 min at 37°C in a moisture chamber. Thereafter, the slides were subjected to a series of washes: 3 times quickly rinsed with H_2_O, 2 × 5 min with TBS-tween and finally 2 × 5 min with TBS. To detect DSBs the slides were thereafter incubated with 0.8 U/μl TdT diluted in 1× TdT buffer (NEB), 0.25 mg/ml BSA, 0.09 mM dTTP, 0.01 mM aminoallyl-dUTP-XX-AF555 and 0.5 mM CoCl_2_ (NEB) for 60 min at 37°C in a moisture chamber. Slides underwent a triple-step wash: 3 times quickly rinsed with H_2_O, 5 min with 10 μg/ml Hoechst 33342 diluted in TBS-tween, 5 min with TBS–tween and finally 2 × 5 min with TBS. The slides were mounted with ProLong Glass antifade (Thermofisher Scientific) and cured overnight before being imaged. All experiments were conducted three independent times and at least three images were acquired per replicate.

### Instruments

Comet-PADDA images were acquired with a Zeiss imager M2 microscope equipped with a Plan-Apochromat 20×/0.8 objective, HXP 120 V light source, Hamamatsu C11440 camera and Zen 2 software (blue edition). Cube filter sets 38HE, 43HE, 49 and 50 were used (all from Zeiss). A minimum of 100 cells were acquired per condition per experiment for the comet-PADDA and several hundred for the on-slide PADDA. Signal strength was enhanced for visualization purposes, but the analyses were performed on original pictures.

Conventional quantification of comet data in the radiation experiment: Comets stained with SYBR Gold (1:10 000) were analysed using a fluorescent microscope (Olympus BX60F-3, Olympus Optical, Japan) and 50 randomly selected comets per slide were scored using an ATV FireWire camera (Stingray, Allied Technologies, Germany).

### Software for image analysis

In all PADDA analyses focusing on the signals from the fluorophores in comet tails showing either SSBs or DSBs, the intensities of fluorescence were analysed using five different modified comet pipelines developed for the software CellProfiler v4.2.4 (cellprofiler.org). Optimized pipelines were used for each experimental setup and the pipelines are included in the Supplementary material. In the radiation experiment, the level of DNA damage in comets was also analysed using the software Comet Assay IV (Perspective Instruments, UK), using the tail intensities (percentage of DNA in the tail) as the indicator of DNA damage.

### Statistical analysis

After testing for normality using Shapiro-Wilks test the integrated intensities that were obtained from the fluorophores in the PADDA experiments were evaluated using either the Mann–Whitney test (H_2_O_2_ experiment and on-slide experiments) or the Kruskal–Walli's test followed by Dunn's multiple comparisons test (radiation, Nt.BsmAI and RsaI experiments). The levels of DNA-damage after electrophoresis using the conventional comet assay (using the Flash comet protocol) were analysed using the Kruskal–Wallis test followed by Dunn's multiple comparisons test (radiation experiment). The level of statistical significance was set to *P* < 0.05.

## Results

### Analysis of SSBs and DSBs

**Figure 1.**
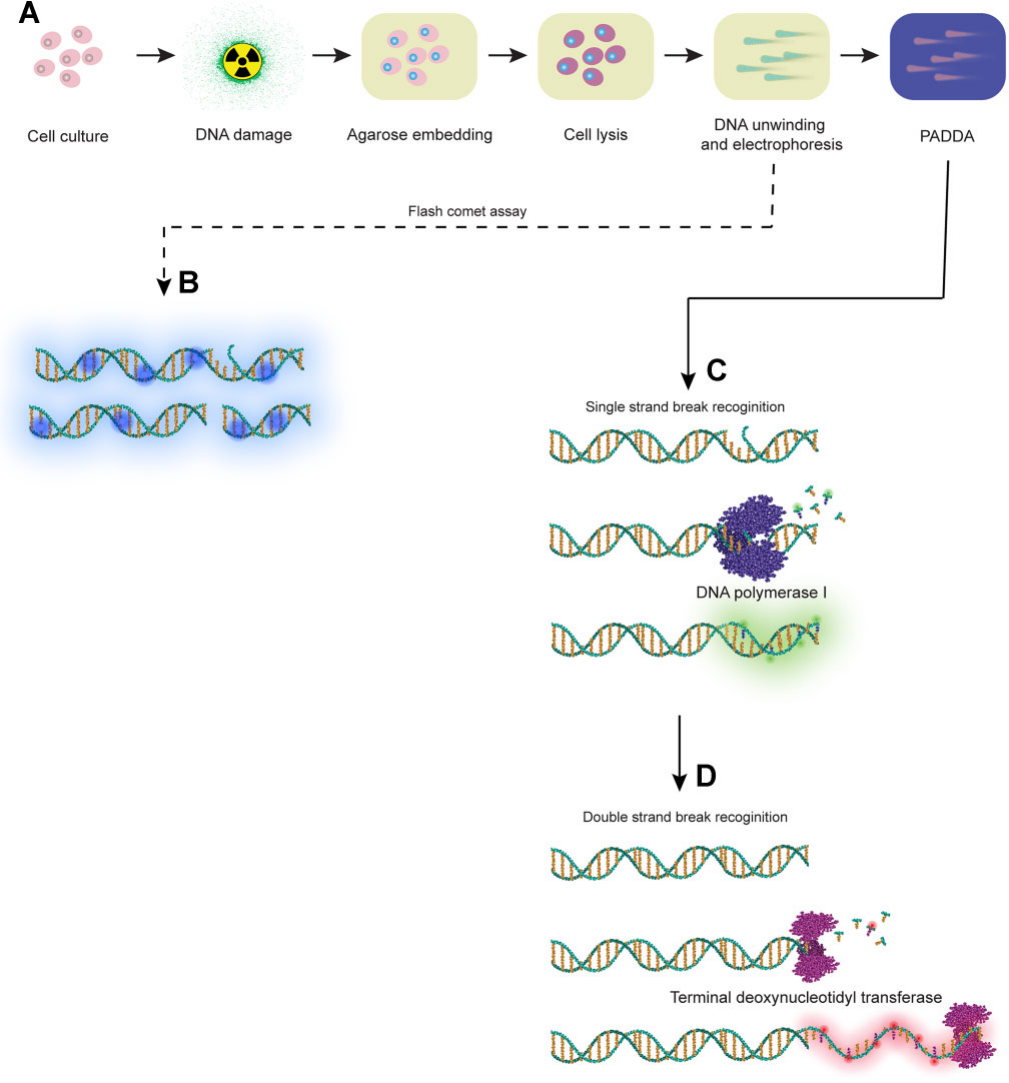
(**A**) A general overview of how the Flash comet protocol is combined with the PADDA-technique. (**B**) When comets are stained in a conventional comet assay, fluorescent stains such as Hoechst 33342 or SYBR Gold are used for a global staining of DNA, not allowing any discrimination between DNA single-strand breaks (SSBs) and double-strand breaks (DSBs). (C, D) SSBs and DSBs are selectively labelled in two steps using the PADDA protocol. In the first step (**C**), SSBs are recognized by DNA polymerase I (Pol I) which starts a template dependent polymerization of new fluorescently labelled nucleotides (fluorophores). This elongation will be terminated by a DSB. As a consequence of this, Pol I will blunt DSBs and thus prime the DSBs for the second step in the PADDA (**D**). The latter step is mediated by the template independent elongation of free 3′OH-groups by terminal deoxynucleotidyl transferase (TdT) which incorporates another fluorophore than the one used in step one, during the elongation in the second step.

In order to selectively visualize SSBs and DSBs in comet heads and tails, we developed a method, PADDA, based upon the sequential labelling of strand breaks, using two different DNA polymerases, i.e. Pol I and TdT. The polymerases will incorporate fluorophore-labeled nucleotides downstream of a DNA break in a template-dependent manner (Pol I) and a template-independent manner (TdT) (17). Hence, the extension product would contain different fluorophores, allowing us to visualize both SSBs (extended by Pol I) and DSBs (extended by TdT) (Figure 1). The protocol utilizes the special features of the enzymes and starts by the dried comets being rehydrated through incubation with Pol I, which will bind to SSBs in the DNA and start a template-dependent polymerization of a new DNA strand with fluorescently labeled nucleotides incorporated and thereafter followed by template-independent elongation of free 3′OH groups by TdT.

In contrast to the staining procedures used in a conventional comet assay, the PADDA labels the DNA (SSBs and DSBs) rather than staining the whole DNA (Figure 1). This means that the signals obtained in the PADDA are specific to break points in the DNA recognized by the different enzymes and the level of fluorescence of the different fluorophores used corresponds to the abundance of either SSBs or DSBs in the cell. The protocol for the PADDA used in the present study, was designed to fit the protocol for the Flash comet assay (Figure 1A). In order to have a global stain for DNA to ensure that the comets could be located, four different conventionally used fluorescent dyes in the comet assay were tested: SYBR Gold, Hoechst 33342, propidium iodide, and acridine orange. The most commonly used stains in the comet assay are either small so-called DNA-intercalators, or so-called minor groove binders to the DNA (36). Both types bind non-covalently to the DNA, but whereas intercalators such as propidium iodide, acridine orange or SYBR Gold, are stacked between DNA base pairs, minor groove binders such as Hoechst 33342, are governed by van der Waals interactions in AT-rich regions (37–39). SYBR Gold is one of the golden standards in the comet assay nowadays, but there is no clear consensus about which stain should be used for visualization of DNA strand breaks in the assay (36,40). We thus tested how four commonly used global DNA stains performed on comets following the PADDA protocol, for this purpose we only monitored DSBs by TdT-mediated incorporation of AF647-labelled dUTP and used unlabelled nucleotides for Pol I.

**Figure 2.**
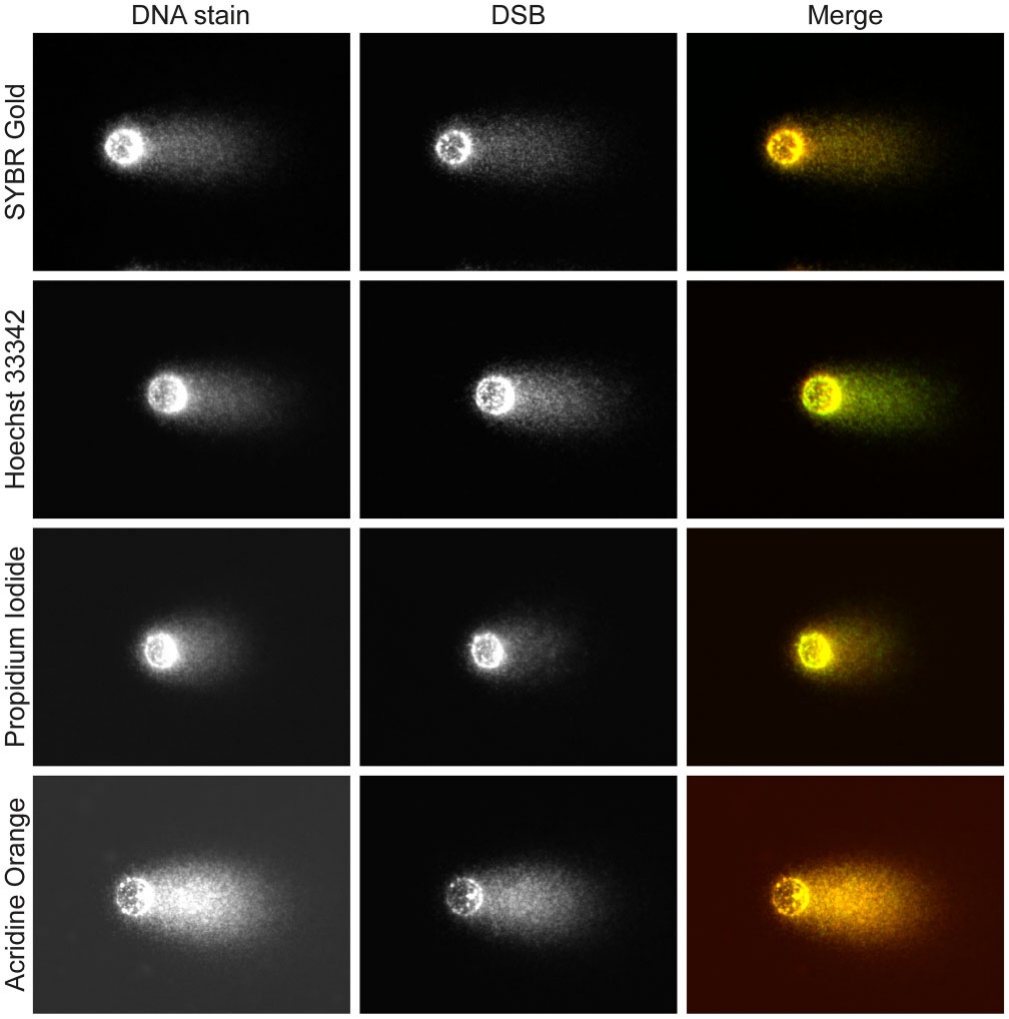
Conventional staining of comets in combination with a selective labelling of DNA-double strands (DSBs, in green): TK6-cells were subjected to single-cell gel electrophoresis using the Flash comet protocol. After the electrophoresis, DSBs were labelled using the PADDA technique where only the fluorophore AF647 was used to label the DSBs. The four different fluorescent stains that were used for the global staining were: SYBR Gold, Hoechst 33342, propidium iodide and acridine orange (all red). In these experiments, the cells had been exposed to 50 μM H_2_O_2_ for 15 min.

No obvious difference in staining pattern was observed for the different DNA stains, except for acridine orange which tended to produce a slightly higher background signal (Figure 2). Since Hoechst 33342 has an excitation and emission profile that do not interfere with conventional fluorophores used in the present study (i.e. AF488, AF555 and AF647), it was used as a global staining of DNA in the PADDA experiments.

### Identification of SSBs and DSBs in comets and comet tails after H_2_O_2_ exposure

**Figure 3.**
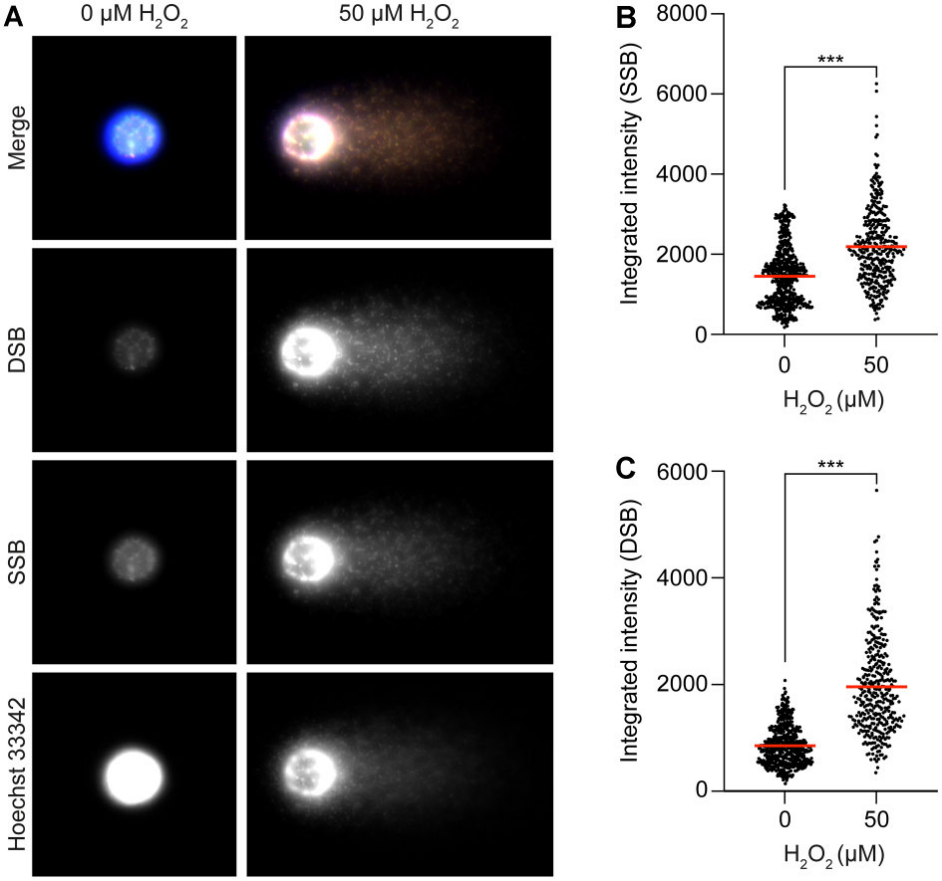
(**A**) shows the labelling of DNA single-strand breaks (SSBs, in green) and double-strand breaks (DSBs, in red) in comets from TK6-cells that had been exposed to vehicle alone or hydrogen peroxide (H_2_O_2_) for 15 min. After exposure, the cells were subjected to single-cell gel electrophoresis using the Flash comet protocol. After the electrophoresis, SSBs and DSBs were labelled using the PADDA technique where the fluorophore AF555 was used to label SSBs and AF647 to label DSBs. Hoechst 33342 (blue) was used for the global staining of DNA. The intensities of the labelled SSBs (**B**) and DSBs (**C**) were quantified using a modified comet assay pipeline for CellProfiler. Mean values for the integrated intensities of SSBs and DSBs are marked with a red line (B, C). A minimum of 100 cells were acquired per condition per experiment. Statistical significance was evaluated using the Mann–Whitney test. ***P < 0.001

To test the performance of the comet-PADDA we selected a few treatments that cause different types of DNA damage. We started with a chemical treatment, H_2_O_2_, known to induce massive DNA damage (15), with an even and random distribution of SSBs and DSBs. The aim of this experiment was to see if it was possible to distinguish between SSBs and DSBs in the comet head and tails using the comet-PADDA. TK6-cells were exposed to either vehicle alone or to a high concentration of H_2_O_2_ (50 μM). High and significant levels of SSBs and DSBs were detected both in the nucleoids and the comet tails (Figure 3A). By measuring the integrated intensities from the fluorophores used to label SSBs or DSBs in the whole comet (head and tail), we were able to visualize more DNA damage than what is normally detected using conventional DNA stains. As expected, most of the SSBs and DSBs signal coincide with the global DNA stain Hoechst, showing a higher abundance of DNA breaks in regions with high DNA content. By doing so it was also possible to visualize the naturally occurring background level of DNA damage in the cells. As expected, the signals from the SSBs and DSBs were found to be significantly increased in the H_2_O_2_-exposed cells (Figure 3B, C).

### Identification of SSBs and DSBs in comets and comet tails after irradiation

**Figure 4.**
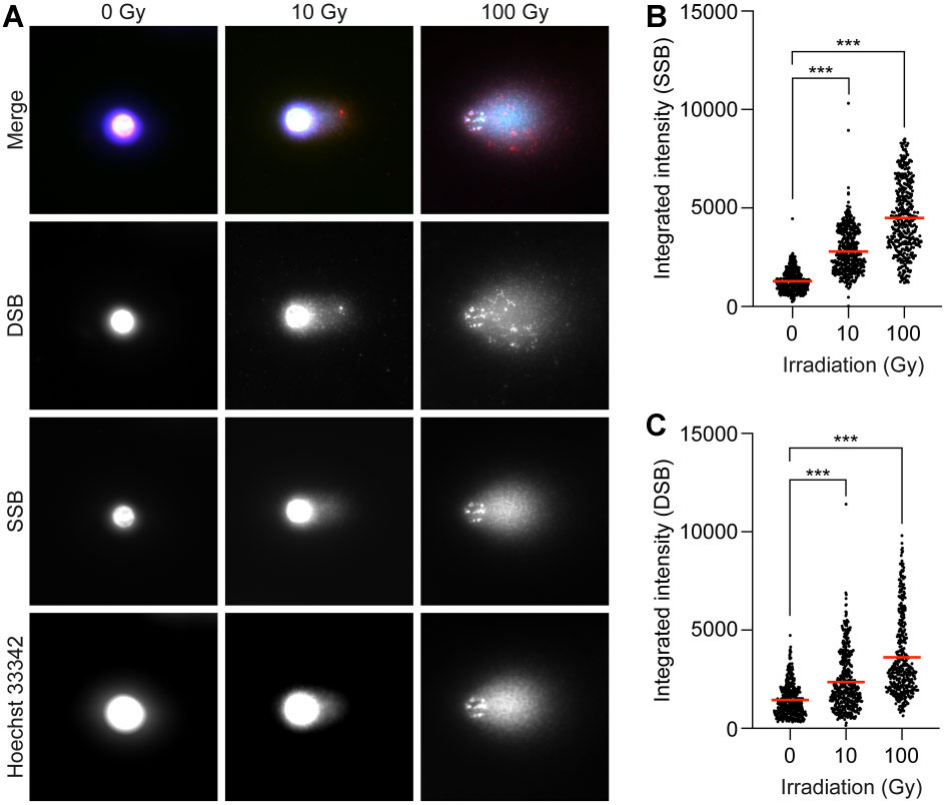
(**A**) shows the labelling of DNA single-strand breaks (SSBs, in green) and double-strand breaks (DSBs, in red) in comets from TK6-cells that had been exposed to vehicle or ionizing radiation from X-rays (0, 10 or 100 Gy). After exposure, the cells were subjected to single-cell gel electrophoresis using the Flash comet protocol. After the electrophoresis, SSBs and DSBs were labelled using the PADDA technique where the fluorophore AF488 was used to label SSBs and AF555 to label DSBs. Hoechst 33342 (Blue) was used for the global staining of DNA. The intensities of the labelled SSBs (**B**) and DSBs (**C**) were quantified using a modified comet assay pipeline for CellProfiler. Mean values for the intensities of SSBs and DSBs are marked with a red line (B, C). A minimum of 100 cells were acquired per condition per experiment. Statistical significance was evaluated using the Kruskal-Wallis test followed by Dunn's multiple comparisons test. ***P < 0.001

After being able to visualize both SSBs and DSBs in the comets in the H_2_O_2_-exposed cells, we next wanted to investigate the signals from SSBs and DSBs in comets from TK6-cell that had been irradiated with a high (10 Gy) or an extremely high radiation dose (100 Gy). As expected, the signals from both the SSBs and the DSBs increased dramatically when the cells were irradiated, especially when the radiation dose was increased from 10 to 100 Gy (Figure 4A). The dramatic increase in DNA damage in the comet tails (a mixture of SSBs and DSBs) was confirmed using the Flash comet protocol with a conventional staining technique (Supplementary Table S1). When the integrated intensities of the SSBs and DSBs in the PADDA were measured, the SSBs and DSBs signals in the comets from the cells that had been exposed to 10 Gy were significantly increased (Figure 4A–C). The proportional increase was similar for both the SSBs and the DSBs. When the radiation dose was increased to 100 Gy, the signal was, as expected, even more dramatically increased for the SSBs and DSBs (Figure 4A–C). Since the proportional increase of the integrated intensities was almost the same for both SSBs and DSBs also at 100 Gy, the SSBs did not seem to have been transformed into DSBs when the radiation dose was increased to 100 Gy. As shown in Figure 4A, the signals from the DSBs seemed to be clustered both in the comet heads and in the comet tails, especially at 100 Gy.

### Identification of SSBs and DSBs in comets from cells treated with Nt.BsmAI

**Figure 5.**
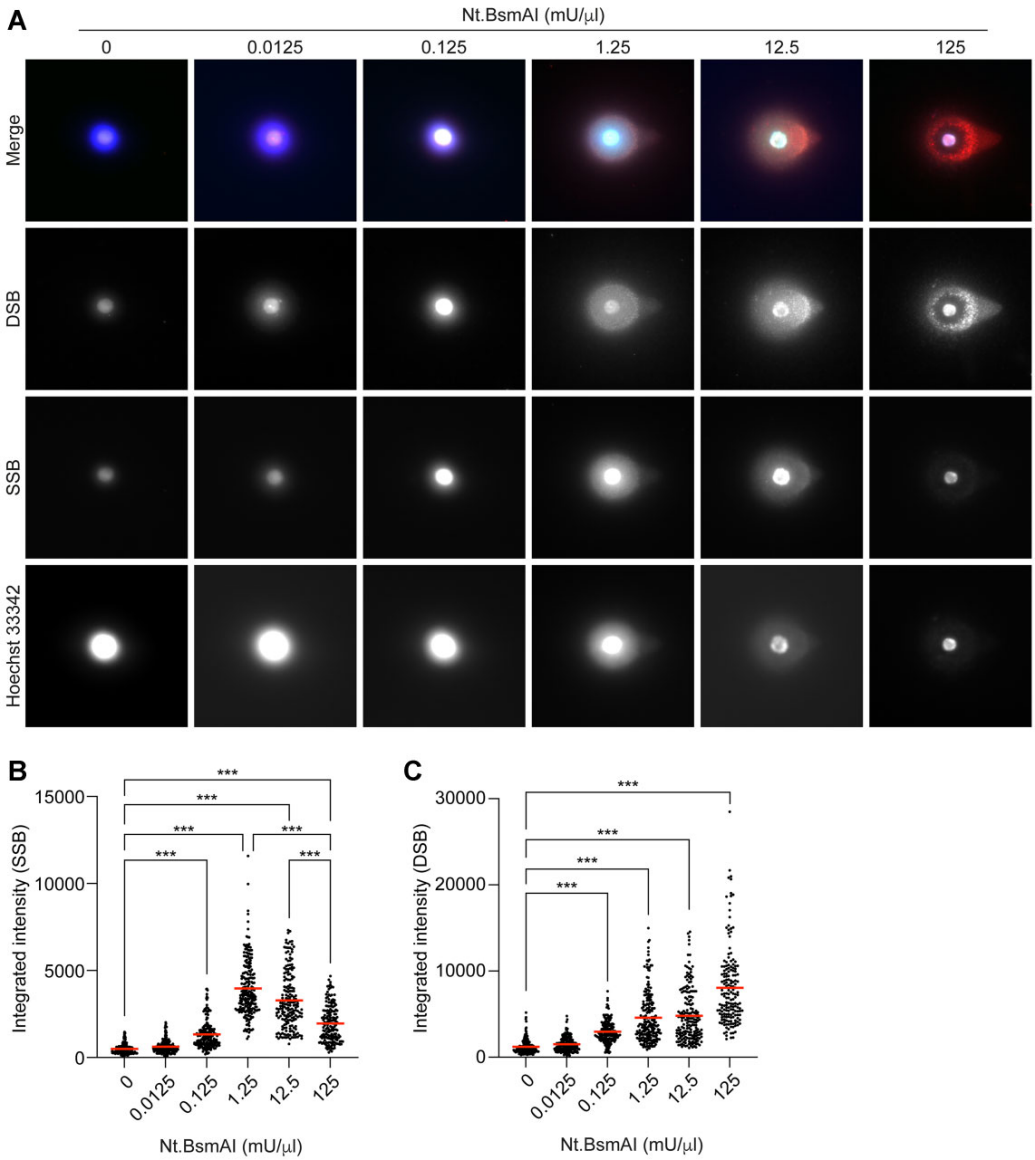
(**A**) shows the labelling of DNA single-strand breaks (SSBs, in green) and double-strand breaks (DSBs, in red) in comets from TK6-cells that had been exposed to vehicle or increasing concentrations of the nickase Nt.BsmAI. After lysis and enzyme reaction, the cells were subjected to single-cell gel electrophoresis using the Flash comet protocol. After the electrophoresis, SSBs and DSBs were labelled using the PADDA technique where the fluorophore AF488 was used to label SSBs and AF555 to label DSBs. Hoechst 33342 (blue) was used for the global staining of DNA. The intensities of the labelled SSBs (**B**) and DSBs (**C**) were quantified using a modified comet assay pipeline for CellProfiler. Mean values for the intensities of SSBs and DSBs are marked with a red line (B, C). A minimum of 100 cells were acquired per condition per experiment. Statistical significance was evaluated using the Kruskal–Wallis test followed by Dunn's multiple comparisons test. ***P < 0.001

Nt.BsmAI belongs to a class of restriction enzymes (nickases) that induce SSBs in double-stranded DNA in a restriction site dependent manner. This means that it will only introduce nicks (SSBs) at specific positions of the genome. However, following alkaline unwinding and electrophoresis all dilutions of Nt.BsmAI used resulted in detectable levels of SSBs and DSBs in the whole comets in an concentration-dependent manner (Figure 5A). Starting from 0.125 mU/μl, the amount of SSBs increased and peaked at 1.25 mU/μl (Figure 5A, B). However, at higher concentrations of Nt.BsmAI, the levels of SSBs started to decline (but still to levels significantly higher than in the vehicle control). In contrast, starting from 0.125 mU/μl Nt.BsmAI, the signals from the DSBs increased steadily in a concentration-dependent manner up to the highest tested concentration (Figure 5A, C). This suggest that many SSBs had been transformed into DSBs at the highest concentrations of Nt.BsmAI, due to the large amount of SSBs induced by Nt.BsmAI to the DNA molecule, which if induced close to each other, and on opposite strands, will result in sticky-end DSBs.

### Identification of SSBs and DSBs in comets from cells treated with RsaI

**Figure 6.**
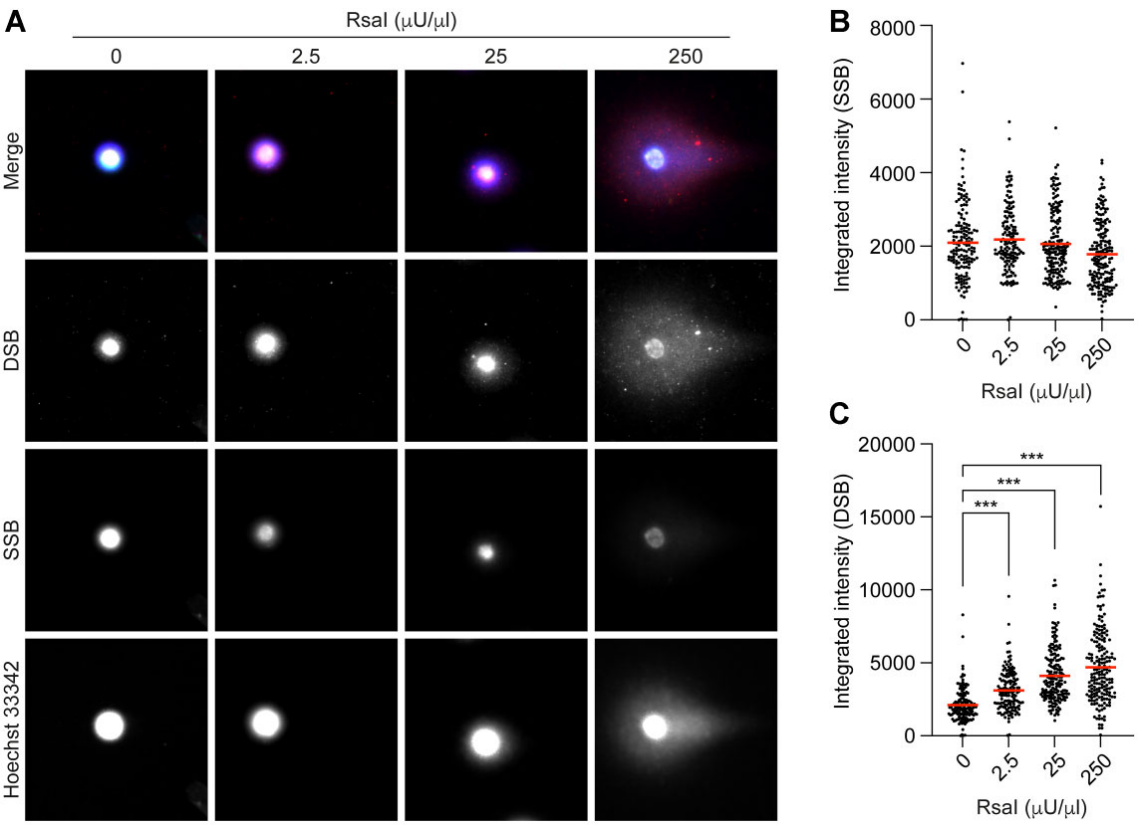
(**A**) shows the labelling of DNA single-strand breaks (SSBs, in green) and double-strand breaks (DSBs, in red) in comets from TK6-cells that had been exposed to vehicle or increasing concentrations of the restriction enzyme RsaI. After lysis and enzyme reaction, the cells were subjected to single-cell gel electrophoresis using the Flash comet protocol. After the electrophoresis, SSBs and DSBs were labelled using the PADDA technique where the fluorophore AF488 was used to label SSBs and AF555 to label DSBs. Hoechst 33342 (blue) was used for the global staining of DNA. The intensities of the labelled SSBs (**B**) and DSBs (**C**) were quantified using a modified comet assay pipeline for CellProfiler. Mean values for the intensities of SSBs and DSBs are marked with a red line (B, C). A minimum of 100 cells were acquired per condition per experiment. Statistical significance was evaluated using the Kruskal–Wallis test followed by Dunn's multiple comparisons test. ***P < 0.001

RsaI is a restriction enzyme that will induce blunt-end DSB into DNA. We tested six different concentrations of RsaI (0 to 25 mU/μl), however, the two highest concentrations fragmentized the genomic DNA to a degree that it was impossible to accurately quantify, so they were omitted from the experiment (Figure 6A). The lower concentrations showed an increased amount of DSBs proportional to an increased concentration of RsaI, which peaked at 250 μU/μl (Figure 6A, C). This was the highest concentration tested which did not fragment the genomic DNA. In contrast to the Nt.BsmAI experiments, which also induced DSBs with increasing concentrations (Figure 5C), RsaI treatment did not induce any significant levels off SSBs at any concentrations tested (Figure 6A, B). This finding supports the notion that after a certain concentration of Nt.BsmAI high amounts of SSBs starts to be converted into DSBs.

### Identification of SSBs and DSBs on fixed cells treated with Nt.BsmAI and RsaI (on-slide)

**Figure 7.**
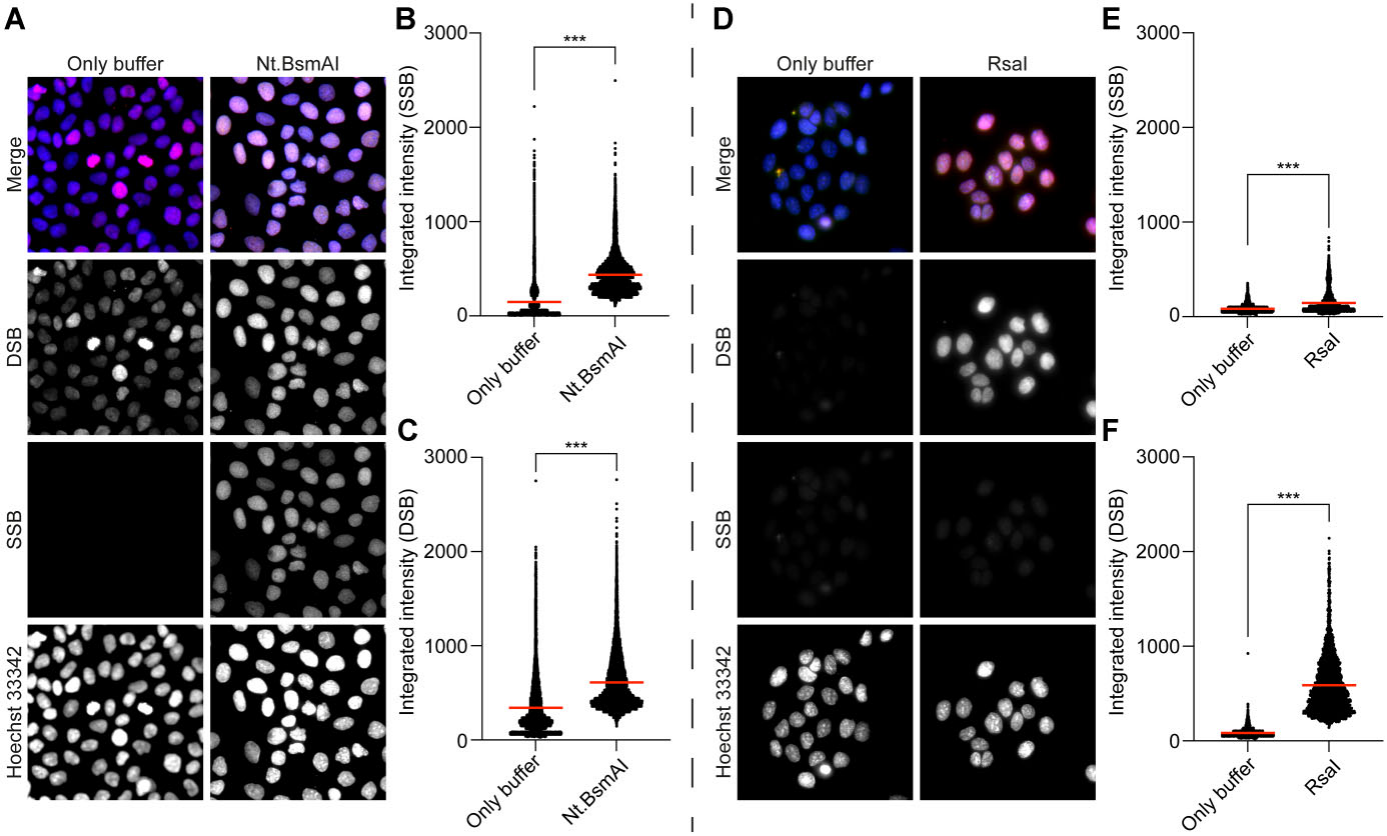
(**A, D**) shows the labelling of DNA single-strand breaks on slide (SSBs, in green) and double-strand breaks (DSBs, in red) in fixed HaCat cells that had been exposed to vehicle, Nt.BsmAI or RsaI. After fixation the cells were exposed to DNA damage treatment and SSBs and DSBs were labelled using the PADDA technique where the fluorophore AF488 was used to label SSBs and AF555 to label DSBs. Hoechst 33342 (blue) was used for the global staining of DNA. The intensities of the labelled SSBs (**B** or **E**) and DSBs (**C** or **F**) were quantified using a modified comet assay pipeline for CellProfiler. Mean values for the intensities of SSBs and DSBs are marked with a red line (B, C, E, F). A minimum of three pictures were acquired per condition per experiment. Statistical significance was evaluated using the Mann–Whitney test. ***P < 0.001

Finally, we wanted to confirm that the Flash comet procedure does not introduce additional damages or denaturation of the DNA. Hence, we investigated how the PADDA performed in adherent fixed cells seeded in chamber slides, which were not subjected to either high pH or electrophoresis. HaCat cells were seeded and fixed with ethanol. After fixation, the slides were treated with 125 mU/μl Nt.BsmAI (Figure 7A) or 25 mU/μl RsaI (Figure 7D). When treating the fixed cells with Nt.BsmAI we were able to detect a distinct increase in SSBs in response to treatment (Figure 7A, B), moreover, we were also able to replicate the results previously seen for the comet-PADDA, where Nt.BsmAI treatment in the higher concentrations gave rise to DSBs (Figures 5C, 7A, C), indicating that it is indeed the high amount of SSBs that after a certain threshold start to transform to DSBs and that it is not the electrophoresis (Flash comet) that induces the DSBs when the nicked DNA is pulled out into the gel. We also tested to treat the slides with RsaI, which induces blunt DSBs. Following exposure to RsaI we were able to detect a drastic increase of DSBs (Figure 7D, F). Moreover, similar to the comet-PADDA, even though statistically significant, we could only see a slight increase in SSBs (Figure 7D, E).

## Discussion

As indicated above, one of the major drawbacks with a conventional comet assay is that it is unable to accurately distinguish between the less hazardous SSBs and more problematic DSBs. This has previously been believed to be achieved by running the comet assay under close to neutral conditions (typically never above pH 10), to monitor DSBs, and then, use an alkaline version of the assay monitoring a mixture of SSBs, DSBs and alkaline labile sites (ALS). Since the image analysis used in a conventional comet assay (under neutral or alkaline conditions) usually is done by staining the DNA with a fluorescent dye, it is impossible to separate SSBs from DSBs, because the signal will be identical regardless of which type of DNA breaks that have occurred. It only provides information about the topological aspects of DNA within the comet, specifically whether it is single or double-stranded, without addressing the actual breakage. The aim with the present paper was to develop a method that would provide information on what types of DNA strand breaks that are present in the comet. We have herein shown that the PADDA is able to selectively visualize SSBs and DSBs in comets.

The method was tested with well-known treatments generating DNA breaks. Hydrogen peroxide, a well-known genotoxic agent was used to induce a mixture of both SSBs and DSBs. As expected, a high dose of 50 μM H_2_O_2_ induced major DNA damage, producing clear and, as most of us doing the comet assay expect, typical comets with heads and tails (Figure 3A). After analyzing comets from H_2_O_2-_treated cells using the comet-PADDA protocol, the signals from the labelling of SSBs and DSBs were significantly increased in comparison to vehicle controls (Figure 3A–C).

An additional and striking finding was that the levels of both SSBs and DSBs in the vehicle controls were surprisingly high, indicating that a relatively high abundance of DNA strand breaks were present inside the comet heads. These must have occurred either before, or during, the electrophoresis (Figure 3A, left panel). The finding that the levels of SSBs and DSBs were relatively high in the negative controls, was confirmed in our radiation, Nt.BsmAI and RsaI experiments (Figures 4–6 and 7). This clearly suggests that the comet-PADDA also allows for detection of SSBs and DSBs in DNA fragments that are too large to be pulled out from the nucleoids during the electrophoresis. This type of strand breaks in the nucleus are missed in a conventional comet assay analysis, since the head of the comet is considered to consist of undamaged DNA. However, the ability to also detect DNA damages in nucleoids may be of great value to consider when it comes to the evaluation of the potential genotoxicity of various compounds especially in regards to the more hazardous DSBs.

When DNA damage was induced by radiation there was a proportional increase in SSBs and DSBs when the radiation dose was increased from 10 to 100 Gy, clearly indicating that the SSBs were not transformed into DSBs even at an extremely high radiation dose. We also found that the DSBs, but not the SSBs seemed to have been clustered both in the comet heads and the comet tails, especially at 100 Gy. This indicates that the larger structures of DNA migrate together, possibly reflecting crosslinked DNA or clustering of large chromatin structures containing multiple DSBs.

The comets obtained in the Nt.BsmAI (nicking) and RsaI experiments had pronounced halos around the comet heads. Typical comet tails were only seen at the highest concentrations of enzyme (Figures 5A and 6A). The halos are most likely the result of the experimental setup for this particular experiment. Under normal conditions in the comet assay, the lysed cells are subjected to alkali unwinding and electrophoresis within minutes after the lysis, but in the Nt.BsmAI and RsaI experiments, the treatment with enzymes had to be conducted after the lysis before the unwinding. This allowed the DNA to diffuse out into the gel creating the halos. Nevertheless, the Nt.BsmAI experiment clearly showed that the PADDA-technique is able to detect SSBs and DSBs separately (Figure 5A). This was particularly evident at the higher concentrations of Nt.BsmAI, where the signals from the labelled SSBs decreased (Figure 5A, B), at the same time the corresponding signals from DSBs increased significantly (Figure 5A, C). As the amount of SSBs increase, and occurring on both strands, these will be converted into sticky-end DSBs. In addition, some of the SSBs will be converted into blunt-end DSBs as Pol I progresses down the DNA strand and encounters an SSB on the template strand. For DSBs with 5′overhangs, the Pol I treatment will incorporate a few nucleotides and generate a blunt-end DSB. During this process there is a possibility for incorporation of fluorophores indicating an SSB. However, as only 1 in 40 nucleotides are fluorophore-labelled, there will not be a substantial level of fluorophores incorporated unless the overhang is very long.

In the RsaI experiment, there was on the other hand a concentration-dependent increase of DSBs, but not of SSBs (Figure 6). Furthermore, for this enzyme, the highest concentrations tested (results not shown) resulted in almost complete degradation of the DNA making it impossible to accurately quantify the DNA damage. That extreme degree of DNA damage is beyond any biological significance that would only be obtained using DNA degrading enzymes in a non-natural *in vitro* situation, such as high levels of aggressive/promiscuous restriction enzymes.

The comet-PADDA is indeed a new way of visualizing DNA damage in the comet assay. Therefore, we decided not to apply the conventional way of performing nor presenting the quantification of comet data. In an effort to provide transparency of the present work, we developed and used five tailored image analysis pipelines based on the software CellProfiler when analysing the data obtained in the comet-PADDA and the on-slide PADDA. These can be found in the Supplementary information. Using these pipelines, the integrated intensity was chosen as the parameter for quantification. The integrated intensity is the total intensity of a signal over a defined region of interest (ROI). It considers both the strength and the spatial extent of the signal, and can be thought of as a measure of the total amount of signal within the ROI. This strategy provides a more complete picture of the overall fluorescence signal while being a less sensitive measurement for background noise compared to the mean intensity of the same ROI.

We have also shown that the PADDA protocol can be used as a stand-alone method on fixed single cells directly on microscope slides, without conducting the comet assay before labelling the SSBs and DSBs with Pol I and TdT (Figure 7). Furthermore, we could also show that the increased DSBs induced by Nt.BsmAI in the comet-PADDA protocol is not due to alkaline unwinding exposing free 3′OH-ends that can be used for non-templated extension by TdT of the DNA, since we obtained the same results by Nt.BsmAI exposure on the fixed cells that have not underwent any alkaline treatments and thus the free 3′OH-ends should belong to DSBs (Figures 5 and 7).

To summarize, in the present paper it has been shown that SSBs and DSBs can be visualized and quantified using the herein described comet-PADDA and on-slide protocol. This made it possible to distinguish between SSBs and DSBs both in comet heads and comet tails in TK6 and HaCat cells that had been subjected to well established chemically, physically or biologically DNA damaging exposures. The method also provides the ability to monitor DNA damages in the comet heads, which cannot be done by conventional comet assays. Although the procedure is a bit more time consuming, the additional level of information will allow for more detailed analysis of DNA damage. Furthermore, the on-slide protocol has made it possible to monitor DNA damage directly in fixed cells. We foresee that the PADDA protocols could be adapted to monitor DNA damage in tissues samples and hence have a future in both pre-clinical toxicity testing as well as in clinical trials.

## Supplementary Material

gkae009_Supplemental_Files

## Data Availability

The data underlying this article will be shared on reasonable request to the corresponding author.
