# Supplementary material for: Visualizing DNA single- and double-strand breaks in the Flash comet assay by DNA polymerase-assisted end-labelling
Source: Nucleic Acids Res. 2024 Jan 23;52(4):e22. doi: 10.1093/nar/gkae009 (PMC10899772; doi:10.1093/nar/gkae009)
Supplement: gkae009_Supplemental_Files [file gkae009_supplemental_files.zip › Table S1.docx]

Supplementary information

Table S1

| Treatment | Radiation dose  (Gy) | Number of analyzed comets | %TDNA  (Mean ± SEM) |
| --- | --- | --- | --- |
| Vehicle | 0 | 100 | 0.99 ± 0.32 |
| X-Rays | 10 | 100 | 24.57 ± 1.1*** |
|  | 100 | 100 | 83.91 ± 0.73*** |

**Table S1** Conventional quantification of radiation-induced DNA damage in comet tails

After TK6-cells had been exposed to vehicle or radiation, they were subjected to single cell gel electrophoresis using the Flash comet protocol. After the electrophoresis, comets were stained with SYBR Gold and analysed using the commercially available software Comet IV. The parameter used for the quantification of DNA damage was the percentage of DNA in the tail (%TDNA). Statistical significance was evaluated using Kruskal-Wallis test followed by Dunn’s multiple comparison test.

***P < 0.001
